# Biopolymer-Based Biomimetic Aerogel for Biomedical Applications

**DOI:** 10.3390/biomimetics9070397

**Published:** 2024-06-30

**Authors:** Yuhan Jeong, Rajkumar Patel, Madhumita Patel

**Affiliations:** 1 Bio-Convergence, Integrated Science and Engineering Division (ISED), Underwood International College, Yonsei University, 85 Songdogwahak-ro, Yeonsugu, Incheon 21938, Republic of Korea; 2 Energy & Environmental Science and Engineering (EESE), Integrated Science and Engineering Division (ISED), Underwood International College, Yonsei University, 85 Songdogwahak-ro, Yeonsugu, Incheon 21938, Republic of Korea; 3 Department of Chemistry and Nanoscience, Ewha Womans University, 52 Ewhayeodae-gil, Seodaemun-gu, Seoul 03760, Republic of Korea

**Keywords:** biopolymers, aerogels, tissue regeneration, wound healing, drug delivery

## Abstract

Aerogels are lightweight and highly porous materials that have been found to have great potential in biomedical research because of some of their unique properties, such as their high surface area, tunable porosity, and biocompatibility. Researchers have been exploring ways to use aerogels to create biomimetic scaffolds inspired by natural extracellular matrices (ECMs) for various biomedical applications. Aerogel scaffolds can serve as three-dimensional (3D) templates for cell growth and tissue regeneration, promoting wound healing and tissue repair. Additionally, aerogel-based scaffolds have great potential in controlled drug delivery systems, where their high surface area and porosity enable the efficient loading and release of therapeutic agents. In this review, we discuss biopolymer-based biomimetic aerogel scaffolds for tissue engineering, drug delivery, and biosensors. Finally, we also discuss the potential directions in the development of aerogel-based biomimetic scaffolds.

## 1. Introduction

Biomimetic principles provide a promising approach for creating high-performance biomaterials. A biomimetic scaffold is a material that not only mimics but also replicates the structure and function of the extracellular matrix (ECM) found in natural tissues [1,2]. This mimicry is crucial for living cells, as it allows for the creation of biomaterials with biomimetic structures and components [3,4]. These biomimetic microstructural scaffolds, by imitating the ECM, increase biocompatibility and enable specific biological functions, resulting in seamless integration with body tissues [5,6,7,8]. Aerogels demonstrate great potential as biomimetic materials in this category, imitating natural structures for various applications.

Aerogel was first developed by Kistler in the early 1930s using a supercritical drying method, and it presented remarkable characteristics [9]. Since then, aerogels have gained much attention and are preferred over hydrogels due to their various characteristics. Most notably, their high porosity and surface area benefit numerous cell functions, such as cell attachment, nutrient diffusion, and drug delivery [10,11]. Furthermore, aerogels do not lose effectiveness in dry environments, making them suitable for certain applications, such as wound healing, where a dry interface can be advantageous [12]. Additionally, hydrogels can swell or shrink significantly depending on hydration levels, whereas aerogels are not as heavily affected.

Aerogels mostly consist of organic and inorganic building blocks, exhibiting a very substantial surface area of open pores, which leads to high porosity and low density overall [13,14]. The typical process for producing aerogels involves drying wet gels. The resulting aerogel solid materials from the drying process are porous and remarkably lightweight, and they are usually made up of 3D cross-linked networks that have some physical similarities to the natural ECM. However, not all dried gels exhibit aerogel characteristics. Porous dry gels can generally be categorized into xerogels and aerogels based on the features of their porous structure during the drying process [15].

Aerogels made of biopolymers have increased in various fields in recent years. Weatherwax and Caulfield prepared the first cellulose-based aerogel in 1971, but it did not receive significant attention due to a large loss of specific surface area [16]. Later, in 2004, Jin et al. successfully prepared a cellulose aerogel with a specific surface area of 160–190 m^2^/g by freeze-drying from calcium thiocyanate tetrahydrate (Ca(SCN)_2_·4H_2_O), a non-derivatizing solvent [17]. Subsequently, biopolymer-based aerogels gained a lot of attention and were developed using various natural macromolecules, such as cellulose, collagen, gelatin, silk fibroin, chitosan, and starch [18,19,20,21,22,23]. The porous microstructure of biopolymer-based aerogels is highly advantageous for absorbing and retaining biological fluids, as well as facilitating fluid and molecule transport. Moreover, biopolymer-based aerogels have the capability to interact with neighboring cells, impacting several cellular processes, like adhesion, proliferation, migration, and differentiation. These aerogels can provide partial mechanical support and a 3D porous structure that promotes homogeneous cell development. Certain specialized aerogel-based biomaterials exhibit positive qualities for biomedical research and can offer biological or chemical/physical cues to direct cell proliferation for applications in tissue engineering and regenerative medicine [24,25,26]. Aerogel’s porous structure, low density, ultrahigh specific area, and easily accessible adsorption sites are also promising for energy storage and wearable sensors [27].

**Scheme 1:**
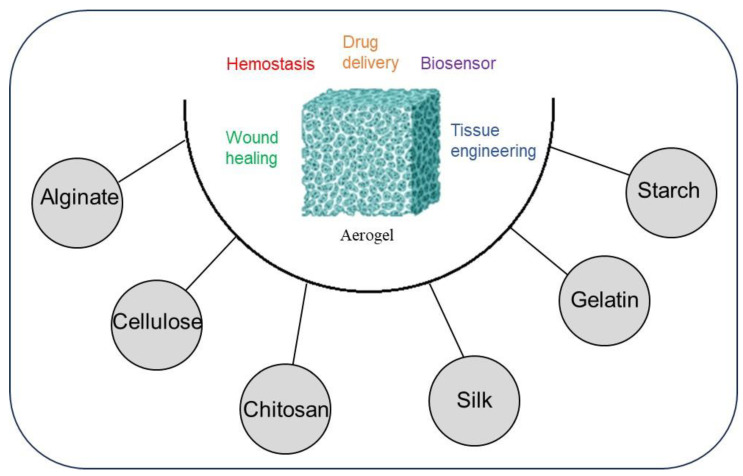
Biopolymer-based aerogel for biomedical applications.

Several review articles have been published on aerogel in the past [13,14,28,29,30]. However, a small number of groups focus on biopolymer aerogel [31,32,33]. Out of them, a few only discussed the chemistry and properties of biopolymer aerogel [32], while others only focused on drug delivery [31], and some reviews concentrated solely on biomedical applications without mentioning biosensors [28,33]. In this review, we discuss all the crucial applications, such as tissue regeneration, wound healing, drug delivery, and biosensors. Moreover, we discuss the recent progress in all important biopolymer aerogels, including cellulose, alginate, gelatin, chitosan, starch, and silk fibroin (Scheme 1).

## 2. Preparation of Biopolymer-Based Aerogel

There are various methods to produce aerogels from biopolymers. However, the most commonly used method involves sol–gel, aging, and drying [13,33]. The sol–gel process creates an aerogel’s 3D porous network structure, while the aging produces a homogenous aerogel, which is defined as network formation and cross-linking. The last phase is solvent removal, referred to as the drying stage or gel-to-aerogel transition.

### 2.1. Sol–Gel Process

The “sol-gel transition” is the stage where a polymeric material dissolves in a solvent to create a gel [34]. Using the right solvent can result in the formation of a homogeneous solution (sol). For example, chitosan can dissolve in an acidic solution [35], while cellulose can be dissolved in an ionic liquid [36]. Sometimes catalysts or crosslinkers are required; for instance, glutaraldehyde can be used to crosslink gelatin. The sol transforms into a gel, creating a three-dimensional network. Various factors can influence this process, such as pH, temperature, chemical composition, additives, and polymer content [37,38].

### 2.2. Aging Process

The aging process is a specific period that enhances the uniformity and strength of the gel’s structure. Typically, controlled humidity and temperature are used during aging [39]. Room temperature may be suitable for certain biopolymers, but slightly higher temperatures may be necessary for others to facilitate network stabilization. Depending on the system, this phase could last for several days or just a few hours. The final qualities of the aerogel can be significantly affected by the duration of the aging processs [40]. Longer aging periods may allow for better network development and stabilization, while shorter times may result in weaker, less uniform gels.

### 2.3. Drying Process

The solvents used in the sol–gel process are completely removed during the drying process. Biopolymer aerogels, with their small pore diameters, large porosities, and delicate skeletons, are highly sensitive to the drying process [32,41]. Different drying techniques, such as supercritical drying [42], freeze-drying [43], and microwave drying [44], are employed in the production of biopolymer-based aerogels. However, each drying method has its own advantages and disadvantages (Table 1).

#### 2.3.1. Supercritical Carbon Dioxide (SC-CO_2_) Drying

Higher-quality aerogels are usually produced using a method called supercritical drying. This method prevents the collapse of the gel structure due to surface tension forces [45]. During this process, the solvent is extracted in a supercritical state, where the pressure and temperature inside the chamber are higher than the critical point of CO_2_. This involves using a supercritical fluid, typically carbon dioxide (CO_2_), to remove the solvent from the gel [46].

#### 2.3.2. Freeze-Drying

Freeze-drying is the most common method, and it works well for biopolymers. Its advantages of low cost, high efficiency, and environmental protection have drawn widespread attention, making it an attractive method of drying [47,48]. The step involves rapidly freezing the gel by immersing it in liquid nitrogen and placing it in a deep freezer. Subsequently, the ice is directly transformed into vapor using a freeze-dryer, bypassing the liquid phase entirely [49].

#### 2.3.3. Microwave Drying

In the field of drying biopolymer-based aerogels, microwave drying is a relatively new approach that offers several advantages over traditional drying techniques [44]. This method aims to maintain the porosity structure of the gel by using microwave radiation to heat and evaporate the solvent inside the gel, resulting in shorter drying times and lower energy consumption [50].

**Table 1:** Advantages and disadvantages of different drying methods.

Method	Advantages	Disadvantages	References
SC-CO_2_ drying	-Produces aerogels with high porosity and minimal shrinkage	-Requires specialized equipment and is more costly	[50,51]
Freeze-drying	-Suitable for heat-sensitive biopolymers and avoids surface tension issues -Environmental friendly and low-cost	-Can result in aerogels with lower mechanical strength and requires careful control of freezing and drying parameters	[52,53]
Microwave drying	-Microwave drying significantly reduces the drying time compared to traditional drying methods	-Tends to lead to large pore formation -Precise control of microwave power and drying time is essential to prevent overheating	[50,54]

## 3. Biopolymer-Based Aerogel

Biopolymer-based aerogels are promising for various applications in biomedicine because of their lightweight and highly porous nature. Biopolymers, like cellulose, chitosan, alginate, gelatin, silk, and starch, are typically used in aerogel production. Cellulose is one of the more common biopolymers for aerogel preparation due to its mechanical qualities and biosafety [55]. Because of its high hydroxyl group content, which promotes the development of strong hydrogen bonds, the molecular structure of cellulose has a high affinity for water and can generate very viscous dispersions even at extremely low solid levels [56,57,58]. On the other hand, alginate and chitosan are often selected specifically for their carboxy and amino functionalities. Aerogels based upon starches are also widely used because of their porous nature and variety of sources [59,60,61]. These materials are naturally biocompatible and non-toxic, making them ideal for medical applications without causing any harmful immune responses. However, these polymers have their own advantages and disadvantages as aerogels (Table 2).

### 3.1. Biopolymer-Based Aerogel in Biomedical Applications

Biopolymer-based aerogels have emerged as promising materials for various biomedical applications due to their unique properties, such as their high porosity, low density, and excellent biocompatibility. These aerogels are derived from natural polymers, making them highly versatile and suitable for various medical uses. We have summarized some of the biopolymer-based aerogel and their applications in Table 3.

### 3.2. Biopolymer-Based Aerogel for Tissue Regeneration

Biopolymer-based aerogels, thanks to their unique properties, have great potential for tissue regeneration. They have a network of interconnected nanopores that closely resemble the ECM found in natural tissues. This porous structure is beneficial because it allows for nutrient diffusion, oxygen exchange, and cell migration, all of which are crucial for tissue regeneration and integration. Furthermore, biopolymer-based aerogels can be engineered to replicate the mechanical properties of native tissues.

A variety of novel materials can be used to create cell-suitable environments. Salecan is an attractive option among these materials, but its complex preparation limits its use. However, a smart aerogel scaffold was developed using salecan and cationic starch (CAS) through ultrasonic-assisted self-assembly without requiring organic or toxic crosslinkers. The ultrasonic waves played a crucial role in enhancing the self-assembly process through ultrasonic cavitation. The aerogel network was created by intense electrostatic interactions between the polysaccharides. Additionally, altering the salecan/CAS ratio enabled accurate control over hydrophilicity, mechanical stiffness, and shape. These aerogels exhibited non-cytotoxic behavior and encouraged connections between cells and the matrix, assisting L929 and 3T3-L1 cells in adhesion and proliferation. Importantly, in vivo experiments demonstrated excellent histocompatibility, with no signs of tissue destruction or abnormal histology in mice treated with aerogels [89]. Aerogels were also found to be useful in delivering signals to biological tissues, enhancing their ability to create cell-suitable environments. To enable electrical signals to be delivered to biological tissues directly, Zamora-Sequeira et al. prepared a nanostructured poly-(3,4-ethylenedioxythiophene) (PEDOT) synthesized using starch/κ-carrageenan aerogel templates. κ-carrageenan biopolymer served as a doping agent to improve electrical responsiveness and biocompatibility [90]. Incorporating κ-carrageenan increased compressive strength by approximately 40% and decreased electrical resistance. Experiments using SH-SY5Y human neuroblastoma cells showed that these cells developed into an implanted neuronal phenotype on the substance. Zein, a biocompatible porogen, was used to induce macroporosity in aerogels made of corn starch. This allowed for creating tailor-made porosities for regenerative medicine without requiring additional leaching steps. The multi-step approach resulted in aerogels with integrated macropores, which offer advantages over conventional methods. Furthermore, SC-CO_2_ treatment effectively sterilizes the aerogels without compromising their mechanical performance. The biocompatibility of the sterile aerogels was promising, with over 80% cell viability observed for aerogels with dual porosity. The study highlights the potential of SC-CO_2_ technology for sterilization and preserving the physicochemical properties of biomaterials, like aerogels, making them suitable for regenerative medicine applications [91].

#### 3.2.1. Bone Tissue Regeneration

Aerogels are favorable for bone regeneration because of their highly interconnected porous nature. Recently, aerogels based on biopolymers have been developed as bone substitutes that offer ideal osseointegration.

For instance, a self-assembled silk fibroin/cellulose interpenetrating network composite aerogel was prepared through freeze-drying of sol–gel, and in situ mineralization showed promise for bone tissue engineering. During the 24-h mineralization process, the scaffold’s mechanical strength increased from 12.7 MPa to 22.4 MPa. The scaffold exhibited an ideal microstructure for bone tissue engineering, high thermal stability, biocompatibility, and up to 99.2% porosity. Additionally, the scaffold increased the growth of human embryonic kidney cells this could be observed due to the mineralized layer growing more cells than the unmineralized surface [69].

**Figure 1:**
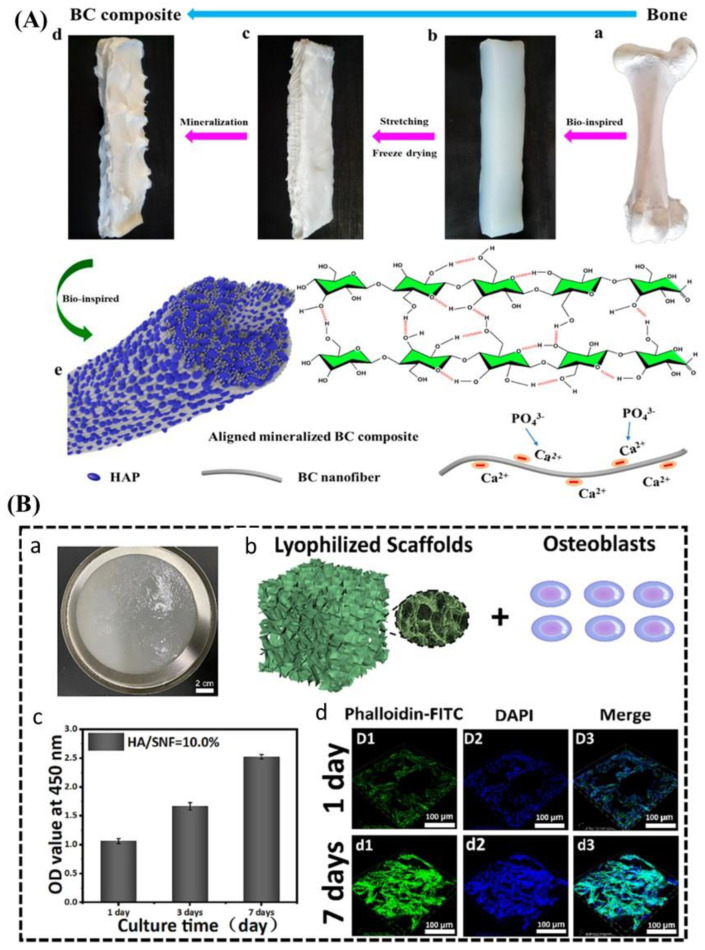
Examples of cellulose and silk fibroin-based aerogel used for bone regeneration. (**A**) A schematic representation of the mineralization process. (**a**) Natural bone; (**b**) BC hydrogel; (**c**) BC aerogel; (**d**) mineralized BC composite; (**e**) microstructure of the AMBC composite. Formation of the HAP mineral particles along the surface of aligned organic BC nanofibers [92]. (**B**) Viability of cells on the HA-assembled SNF aerogel scaffolds. (**a**) Photographs of the wet aerogel scaffolds; (**b**) the HA-assembled SNF aerogel was used as a 3D scaffold for the cell culture; (**c**) an assessment of the osteoblasts’ cell viability; (**d**) confocal images of the osteoblasts for 1 and 7 days. Scale bars: 100 μm [94]. BC, bacterial cellulose; AMBC, aligned and mineralized bacterial cellulose; HA, hyaluronic acid; SNF, silk nanofibers.

Combining biopolymer-based aerogels with the inorganic skeleton components can create bone repair materials. Iglesias-Mejuto et al. prepared a unique uncontaminated cellulose-in-cellulose aerogel incorporating superparamagnetic iron oxide nanoparticles (SPIONs) through an integrated platform of 3D printing and SC-CO_2_ technologies [70]. Methylcellulose (MC) and bacterial nanocellulose (BC) were utilized to create aerogels with high structural resolution and hierarchical porosity. These SPION-doped aerogels showed magnetic properties, which had no detrimental effects on NIH/3T3 cell viability, hemolytic activity, or the mortality of A. salina. Additionally, scCO_2_-based sterilization and long-term storage did not affect the aerogels’ physicochemical performance, confirming their potential for use in bone tissue engineering. These results demonstrated that aerogels based on cellulose are suitable for bone tissue engineering, particularly with the potential for unique magnetic properties offered by SPIONs. In another example of aerogel-based biomimetics, Cheng et al. presented a bioinspired approach to mineralize aligned BC using CaCl_2_ and K_2_HPO_4_ solutions, mimicking the natural bone structures [92]. The aligned cellulose nanofibers provide a template for the uniform inclusion of hydroxyapatite (HAP) with a high mineral content. The resulting aligned and mineralized bacterial cellulose (AMBC) composite has a hardness of 0.37 ± 0.18 GPa and an elastic modulus of 10.91 ± 3.26 GPa, demonstrating remarkable mechanical strength. In comparison to mineralized bacterial cellulose that is not aligned (NMBC), AMBC has an elastic modulus of 210% higher and a hardness value of 95% higher. This biomimetic mineralization approach offers a promising strategy for developing bone substitutes with excellent mechanical properties (Figure 1A).

The inorganic component in biomaterial-based aerogel has also been shown to be an excellent implantable substance for bone tissue engineering, which can aid in tissue regeneration and subsequent cancer treatment. A study by Al-Jawuschi introduced a new method of creating a 3D aerogel-based composite scaffold with a dual-network structure. The silk fibroin methacrylate (SF-MA) scaffold showed photothermal conversion properties due to the NIR absorption of integrated Bi_2_S_3_ nanobelts. The scaffold heat was generated upon laser irradiation, which triggers controlled drug release and effectively ablates cancer cells that adhere to the scaffold. The scaffolds also exhibit excellent biodegradability, making them suitable for tissue regeneration after removing cancer cells. These multifunctional 3D-printed composite aerogel scaffolds hold promise for regenerative tissue in bone tissue engineering and subsequent cancer therapy [71]. Furthermore, SF-MA with methacrylate hollow mesoporous silica microcapsules (HMSC-MA), showed enhanced mechanical stability, cellular ingrowth and proliferation, and osteoblastic differentiation when printed as a composite gel. These scaffolds have high osteo differentiation markers, such as ALP, OPN, and mineralization signals. The resulting scaffolds demonstrated improved mechanical stability due to photo-crosslinking during printing and further interconnectivity from freeze-casting, facilitating cell infiltration and growth. The incorporation of HMSC also promoted antibacterial drug delivery [72].

Another recent study developed 3D scaffolds made of biocompatible silica–silk fibroin for the purpose of bone tissue engineering. A mixture of surface modification, micro-extrusion-based 3D printing, sol–gel and self-assembly procedures, and directional freeze-casting/drying techniques were used to create the scaffolds. These were modified via thiol-ended antimicrobial and cell adhesive peptide sequence (SH-CM-RGD) conjugation, which improved the bactericidal qualities and responsiveness of the cells. This hybrid scaffold had hierarchically organized porosities, potent bactericidal efficiency against bacteria, and enhanced osteoblast cell growth and proliferation. The scaffolds showed promise for bone defect repair, offering anti-infective and osteoconductive properties for enhanced bone tissue engineering applications [93].

Silk-based aerogels have multiple applications as scaffoldings, including in a nanofibrous scaffold made up of silk protein nanofibers (SNFs) and hyaluronic acid (HA) for tissue regeneration. HA enhanced SNFs’ hydrophilicity and bioactivity by acting as a bioglue, resulting in aerogel scaffolds utilizing SNFs that showed high porosity, natural bioactivity, and stable structures. These scaffolds mimic the extracellular matrix’s composition and hierarchical structure, providing an ideal osteoblast cell adhesion and proliferation microenvironment. With HA assistance, the SNF scaffolds kept their shape for more than three weeks in protease/hyaluronidase solutions, demonstrating their structural stability. This biomimetic scaffold improved osteoblast cell function and growth through enhanced mechanical properties and biological functionality (Figure 1B) [94]. Similarly, SNFs biomimetic aerogel using polysaccharide-based assembly methods for bone tissue engineering applications. Natural polysaccharides, such as CS, HA, and SA, regulate SNF assembly and provide stability and tunable mechanical properties to the scaffolds. Regulating polysaccharide content achieved ultrahigh porosity, allowing for better cell viability. SNF with chitosan aerogels showed excellent biocompatibility and functionality for osteoblast cells. The SNF aerogel scaffold that was built by CS and submerged in SBF solution had the most mineralization, as well as strong bone marrow stem cell (BMSC) cell adherence and total cell survival [95].

Starch-based aerogels also have a place in tissue scaffolding similar to silk or cellulose. In a study, Goimil et al. developed PCL scaffolds for bone regeneration using starch aerogel microspheres and ketoprofen, a bioactive compound [73]. The starch aerogel microspheres the size of a micron improved the scaffold’s porosity and pore interconnectivity and encouraged bone tissue formation. The scaffolds exhibited sustained ketoprofen release, making them suitable for scaffolds loaded with drugs whilst having precise pore structures for cell ingrowth and controlled drug release. The less toxic conditions used to process the scaffolding also open possibilities for incorporating other compounds to promote cell growth for better healing processes. Whilst aerogels exhibit high mesoporosity, they lack macroporosity. A study by Martins et al. used a carbon dioxide-induced gelation method to create dual meso- and macroporosity hybrid alginate–starch aerogels that are crosslinked with calcium [74]. By adjusting the expansion rate during gelation, the macroporosity of the aerogels was significantly increased from 2 to 25% while retaining mesoporosity. The starch-augmented aerogels also had sufficient water uptake and an increased Young’s modulus. In vitro tests showed that the aerogels exhibited bioactivity, generating hydroxyapatite crystals when subjected to artificial bodily fluid; this phenomenon was ascribed to the calcium content of the matrix. These findings highlight the promising characteristics of alginate–starch aerogels for use in regenerative medicine, particularly in bone tissue engineering.

**Figure 2:**
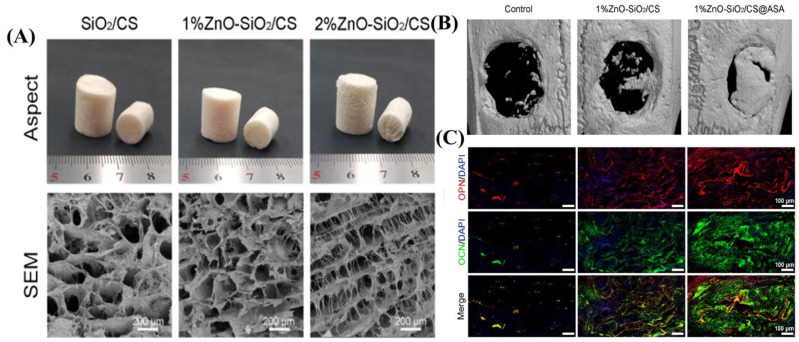
(**A**) macrophotographs and SEM images of different aerogel scaffolds, (**B**) microcomputed tomography (Micro-CT) photographs of a rat model of cranial defects in vivo up to 12 weeks, and (**C**) immunofluorescent staining including OPN (red), OCN (green), and nuclei with DAPI (blue) complex staining [96].

Some other biopolymers, such as alginate, gelatin, and chitosan-based aerogels, have been extensively used in bone regeneration [85,86,96]. In a study by Iglesias-Mejuto et al., 3D-printed alginate aerogel demonstrated bone regeneration [85]. The unique combination of 3D printing and aerogel technology enhanced the architecture and performance of the scaffold. The scaffold was created by printing alginate–hydroxyapatite (HA) hydrogels in three dimensions and then drying them in supercritical CO_2_. The resulting scaffolds exhibited high porosity, biocompatibility, and fidelity to the initial CAD design. The porosity decreased with the HA content while promoting cell survival, adhesion, and migration, especially for mesenchymal stem cells (MSCs) and fibroblasts. The enhanced fibroblast migration to injured sites accelerated bone regeneration. Recent research found that nanofiber aerogels have the potential to be used in tissue regeneration due to their biomimetic properties and highly porous structure. Xhao et al. recently created ZnO-SiO_2_ nanofibers that showed promising results for bone regeneration when combined with chitosan (CS) and aspirin (ASA) [96]. The 1%ZnO-SiO_2_/CS@ASA scaffolds showed excellent biocompatibility, bioactivity, anti-inflammatory properties, and mechanical strength. It also significantly enhanced vascularization and osteoblast differentiation in vitro. In a mouse cranial defect model, these scaffolds led to a much higher rate of osteogenesis (22.76 ± 1.83%) compared to the control group (5.59 ± 2.08%). The positive effects are attributed to releasing Si^4+^, Zn^2+^, and ASA from the scaffolds, suggesting an effective strategy for treating bone defects (Figure 2). A scaffold with electroactive properties was developed by combining a nanofiber aerogel scaffold with polyhydroxybutyrate (PHB) and zinc oxide (PHB@ZnO) nanofibers. This scaffold showed strong potential for promoting tissue repair and regeneration by creating an electrical microenvironment that supports bone healing. When activated by ultrasound stimulation, the PHB@ZnO nanofibers with CS further enhanced adhesion, migration, osteogenic differentiation, and bone formation. It upregulated the signaling pathway of the calmodulin –calcineurin–nuclear factor of T cells. After being implanted into a rat shin bone defect model for 8 weeks, the scaffold achieved a high bone volume/tissue volume fraction of 66%, demonstrating its potential to significantly accelerate bone repair [97]. Furthermore, fibrous aerogel is designed with a gradient composition that mimics cartilage-to-bone transition. In a study by Zhang et al., a novel 3D fibrous gradient aerogel (A-G) was designed to mimic the transition from cartilage to bone [86]. The aerogel features three layers: the top layer contains glycosaminoglycan achieved by poly(_L_-lactide) (PLA)/gelatin (Gel)/hyaluronic acid/chondroitin sulfate composite fibers, while the middle and bottom layers contain apatite achieved by PLA/gel fibers. When implanted in rabbit knees for 12 weeks, the cell affinity peptide (E7-peptide) grafted aerogel (A-E7G) significantly outperformed the osteochondral interface, showing a mature structure and clear tidemark. This success is attributed to the incorporation of glycosaminoglycans (HA and CS) and mineral components into the scaffold fibers, as well as the grafting of E7-peptide for enhanced cell affinity.

#### 3.2.2. Cartilage Tissue Regeneration

Similar to bone regeneration, an optimal scaffold must have a few key features for cartilage regeneration via tissue engineering to be successful. Among these qualities are a biomimetic three-dimensional (3D) architecture that promotes cell adhesion, the right amount of porosity to encourage cell ingrowth, enough mechanical strength to keep the structure in place, strong biocompatibility, and biodegradability. Aerogel is a scaffold used for cartilage repair applications [75,76,86]. However, few papers discuss using aerogel-based materials for cartilage tissue regeneration compared to bone replacement.

Scaffolds for tissue engineering utilizing an accurate Poisson’s ratio (PR) are crucial in replicating native tissue environments to enhance cell survival and function. Therefore, it is essential to easily adjust the geometrical parameters to import calculated ratios for tissue engineering purposes. Sun et al. fabricated a composite poly(ethylene glycol) diacrylate/cellulose nanofibril aerogel scaffold using COMSOL simulation to regulate the PR [75]. Stereolithography and freeze-drying techniques created nine scaffold groups with PRs ranging from −0.5 to 0.85. The scaffolds with zero PR showed the highest collagen II marker gene expression, making them the most conducive for bone marrow mesenchymal stem cell (BMSC) differentiation into chondrocytes. The study provides insights into scaffold design for cartilage repair and other tissue engineering scaffolds. Using stereolithography (SLA) and freeze-drying, cellulose nanofibers (CNFs) and polyethylene glycol diacrylate (PEGDA) were combined to form CNF/PEGDA aerogels with multiscale pore architectures. The aerogels showed a random distribution of micropores and a uniform macropore network topology. SLA enables customization of macropore structure, resulting in aerogels with positive, negative, or zero PR, offering a wide variety of mechanical options. Further tests of mechanical strength and chondrogenic induction demonstrated the potential of the negative PR scaffold to promote mouse BMSC proliferation and chondrogenic differentiation, suggesting a promising avenue for cartilage repair in tissue engineering [76].

### 3.3. Biopolymer-Based Aerogel for Wound Healing and Hemostat Application

The process of wound healing is highly intricate, involving various cellular, biochemical, and physiological activities that can be affected by internal and external factors, such as wound moisture, oxygenation, infection, and maceration. Recent studies suggest that aerogels hold great promise for wound healing applications due to their high fluid retention capacity, excellent mechanical properties, and ability to minimize arterial blood loss [98,99]. Biopolymer-based aerogels, in particular, have garnered significant attention in biomedical applications in part because of their abundance, biocompatibility, and biodegradability [77,100,101]. Moreover, they have demonstrated exceptional hemostatic properties, promoting platelet activation and preventing blood loss, as reported in some recent studies [102,103].

**Figure 3:**
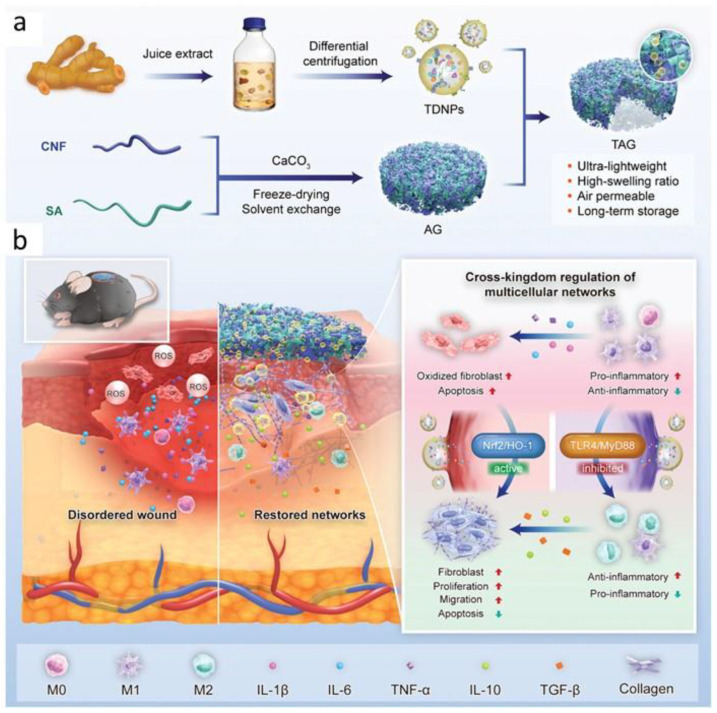
TDNP-loaded cellulose aerogel functions as an immunomodulatory dressing for diabetic wound healing by modifying multicellular networks; (**a**) isolating TDNPs and creating AG and TAG dressings; (**b**) TAG promotes diabetic wound healing by increasing antioxidant capacity, reducing inflammation, and reestablishing multicellular regulatory networks in the wound microenvironment [78]. TDNPs, turmeric-derived nanoparticles; AG, aerogel; TAG, TDNPs-loaded aerogel.

Researchers utilized oxidized bacterial cellulose (OBC) and platelet extracellular vesicles (pVEs) to develop an ultralight hemostatic aerogel that acts as a physical barrier. The aerogel quickens the production of clots, concentrates both platelets and clotting factors, and absorbs blood. In vivo experiments showed that it reduces blood loss and bleeding times and promotes wound healing in rat liver and mousetail. It also showed wound healing in full-thickness skin defects. This work offers a rapid and effective treatment for challenging wounds and highlights the potential for aerogel-based techniques in this field [77]. Other examples of wound dressings utilizing cellulose-based aerogels include a turmeric method. A new method for managing diabetic wounds involves using turmeric-derived nanoparticles (TDNPs) loaded into aerogel dressings (TAG) that target the wound microenvironment. In vitro experiments showed that TDNPs can effectively minimize inflammation, eliminate reactive oxygen species (ROS), and promote healing in fibroblasts and macrophages. The developed TAG has excellent biocompatibility, controlled TDNP release, and water absorption capacity (Figure 3). In vivo, evaluation using a diabetic mouse model confirmed TAG’s effectiveness in facilitating diabetic wound healing. This approach holds promise for enhancing diabetic wound treatment by overcoming the limitations of conventional therapies and biologically based treatments [78]. Starch-based aerogels are also great for hemostasis and wound management. Li et al. introduced a new dressing called a hierarchical porous structure (HPS), designed for wound management and efficient hemostasis [79]. The dressing comprises a non-woven substrate, konjac glucomannan (KGM) aerogel, and bi-functional microporous starch (BMS). The first level of pores are provided by the non-woven fabric, the second level by the KGM aerogel, and the third level by the BMS-inlaid hydrophilic pores. All in all, this creates a triple porous structure that quickly captures red blood cells and platelets, enhancing its hemostatic abilities. Animal experiments have demonstrated the dressing’s ability to stop bleeding from the ear artery and liver in short periods (97.6 ± 15.2 s and 67.8 ± 5.4 s, respectively). The dressings also promote wound healing in two weeks and contain antimicrobial qualities. The HPS dressing is a promising solution for managing wounds and providing both hemostatic and wound healing functionalities in a single dressing.

**Figure 4:**
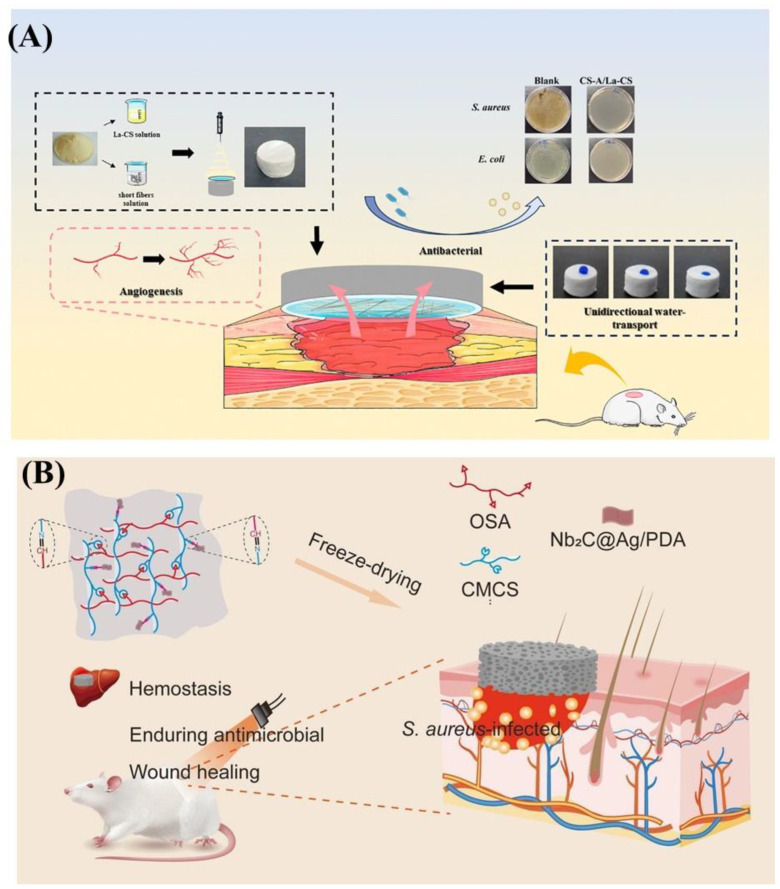
Schematic illustration of the (**A**) chitosan aerogel preparation and its clinical wound care [104], and (**B**) sodium alginate oxide and carboxymethyl chitosan preparation and its application for infected wounds [105].

Excessive hydration of a wound can lead to increased inflammation and damage to the tissue around the wound, which can delay the healing process. Researchers have developed a chitosan nanofiber aerogel with unidirectional water-transport ability as a wound dressing to address this issue. This dressing consists of a hydrophilic chitosan aerogel (CS-A) outer layer and a hydrophobic laurylated chitosan (La-CS) nanofibrous membrane inner layer as the Janus dressing. This design allows for excellent liquid absorption (2987.8 ± 123.5%), air and moisture permeability (997.8 ± 23.1 g/m^2^/day), and mechanical strength (5.1 ± 2.6 MPa). The dressing showed good porosity, effective unidirectional water transport, and elasticity, which are all essential for wound healing. Additionally, it has demonstrated strong antibacterial properties due to the biological activity of chitosan. In animal studies, the dressing reduced the inflammatory phase, promoted blood vessel formation, and accelerated collagen deposition, leading to faster wound healing (Figure 4A) [104]. Furthermore, an antibacterial dressing was prepared by Li et al. using MXene-based polysaccharide composite aerogel [105]. The researchers used sodium alginate oxide (OSA), carboxymethyl chitosan (CMCS), and Nb2C@Ag/PDA (NAP) nanoparticles to create the OSA/CMCS-Nb2C@Ag/PDA (OC/NAP) composite aerogel. The aerogel exhibited outstanding swelling properties, biocompatibility, porosity, and sustained antimicrobial efficacy. Antimicrobial tests revealed that the OC/NAP composite aerogel maintained nearly 100% inhibition of *S. aureus* and *E. coli* for over 25 h in vitro and 14 days in vivo. Additionally, in vitro and in vivo hemostatic tests indicated excellent hemostatic properties (Figure 4B). To improve the antibacterial properties of wound dressings, Karan et al. used copper–cystine-based high aspect ratio structures (CuHARS) combined with electrospun poly(ε-caprolactone)/gelatin (PCL/gelatin) nanofiber aerogels [106]. The release of Cu^2+^ from CuHARS at specific concentrations catalyzed the production of nitric oxide (NO) from S-nitrosothiols (RSNO) in human blood. This process promoted angiogenesis and endothelial cell migration, facilitating successful lumen development and the formation of blood vessels in wounds. Additionally, the combined effect of Cu^2+^ and NO showed a synergistic impact in eradicating methicillin-resistant *S. aureus* (MRSA). Nanofiber aerogels with precise microchannels and the LL-37-mimic peptide W379 have enhanced diabetic wound healing. In vivo studies show that these aerogels significantly promote cell penetration, improving cell infiltration, neovascularization, and re-epithelialization in diabetic wounds. The rapid re-epithelialization is attributed to phospho-extracellular signal-regulated kinase (p38 MAPK) upregulation after W379 treatment [88]. The chitosan-gelatin-dopamine-vascular endothelial growth factor (CG-DA-VEGF) aerogel has also been developed to support wound healing processes. The CG-DA-VEGF aerogel was developed using an environmentally friendly freeze-drying process, employing chitosan and dopamine-grafted gelatin as base materials. EDC/NHS serve as non-toxic crosslinking agents. This porosity enables the aerogel to support the biological processes necessary for wound healing. Animal studies demonstrate that this aerogel significantly improves angiogenesis, re-epithelialization, and collagen deposition, leading to a nearly 100% wound healing rate within 14 days [87].

**Figure 5:**
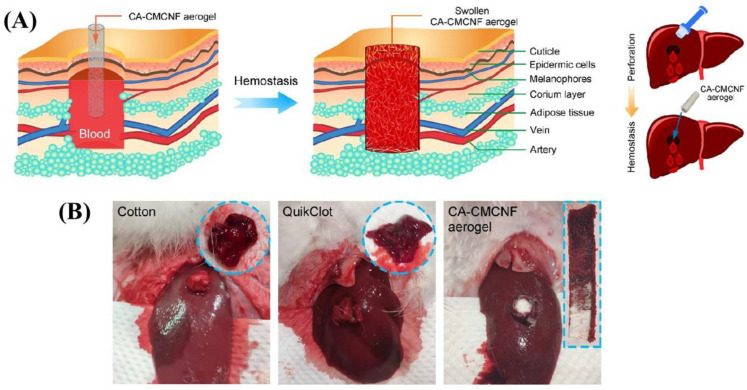
Hemostasis in a liver-penetrating wound model; (**A**) schematic of CA-CMCNF aerogel halting bleeding in a rabbit liver-penetrating wound model; (**B**) Pictures of hemostatic agents halting bleeding from lesions that penetrate the liver of rabbits [101]. CA-CMCNF, citric acid cross-linked carboxymethyl cellulose nanofibers.

There are many opportunities within the field of hemostat material study to control hemorrhages. A recent study has shown a novel hemostat for noncompressible hemorrhage control, which is made up of a citric acid (CA)-crosslinked and N-hydroxysuccinimide (NHS) ester-activated carboxymethyl cellulose (CMC-NHS) aerogel. The CA strengthens the CMC, while the NHS promotes adhesion to wounded tissue and increases blood coagulation. CMC dissolves at the adhesion interface, allowing for easy removal. The aerogel quickly adheres to wounds, demonstrating robust mechanical strength, rapid hemostasis, reduced blood loss, good biocompatibility, and antibacterial properties. This innovative approach may inspire the development of additional hemostats with strong adhesion and easy detachment using aerogels and CMC-NHS [100]. Another citric acid-cross-linked carboxymethyl cellulose nanofiber (CA-CMCNF) aerogel showed a potential solution for hemorrhages. It expands quickly upon contact with water, filling wound cavities and applying pressure to stop bleeding. The aerogel’s carboxyl groups promote active coagulation pathways, improving its hemostatic capability. Additionally, it is biocompatible and has strong antibacterial properties. The biocompatible parts of CMCNFs and CA are used in a straightforward lyophilization and heating process to create the aerogel. The CA cross-links CMCNFs and protonates carboxyl groups, forming double hydrogen bonding within the walls of the aerogel’s microchannels, which allows better water penetration. This is crucial for the aerogel’s ease of self-expansion, aiding in its hemostatic properties (Figure 5) [101]. Neovascularization is critical in wound healing, impacting the quality of neotissue formation. A biphasic gel comprising a highly porous aerogel and a degradable fibrin hydrogel has been developed to promote vascularization in implanted cell-containing grafts. The aerogel, consisting of cellulose and polyethylene short microfibers, is freeze-dried and can be shaped using 3D printing or mold-based techniques. Combined with fibrin hydrogel, this composite facilitates cellular ingrowth and vascularization. In contrast to the control (commercial surgical felt/fibrin), the biphasic gel doubled vascular ingrowth with deeper penetration after subcutaneous implantation in mice. Additionally, the biphasic gel exhibited higher vascular endothelial markers (CD 31) and vascular smooth muscle markers (αSMA) after four weeks [107]. Gelatin has excellent solubility at the 37 °C tissue surface but lacks the ability to form a strong physical barrier for wound management and hemostasis. Researchers developed a gelatin-based derivative (Ged) that swells upon absorbing the tissue hydrate layer to address this limitation. This modification resulted in Ged exhibiting excellent adhesion properties and long-term swelling at 37 °C. Active ingredients from the Bletilla striata complex (Bscai) were added to create the Bscai/Ged hemostatic material, which has shown a rapid hydrate layer trigger rate, strong wet tissue adhesive qualities, outstanding hemostatic properties, reduced inflammatory responses, accelerated wound healing, promoted collagen deposition, and enhanced angiogenesis in vivo experiments [108].

### 3.4. Biopolymer-Based Aerogel for Drug Delivery

Some of the aerogels’ physical properties appear to be very advantageous for drug delivery. Depending on the stage of the production process, drug loading into the aerogel structures might happen in the sol–gel mixture, during the solvent exchange, or during the supercritical fluid (SCF) drying. Recently, several drug delivery aerogel compositions have been studied and suggested for various uses.

Groult et al. investigated the release of theophylline from cellulose-pectin composite aerogels prepared by SCCO_2_ [109]. Varying the cellulose and pectin concentrations and calcium content resulted in changes to the aerogel properties. The release mechanisms varied with pectin content, and crosslinking pectin with calcium extended drug release time. The aerogel-loaded theophylline was released in vitro into the simulated gastric fluid (SGF) and then into the simulated intestinal fluid (SIF). The findings suggest the potential for tailored drug release kinetics in bio-based porous materials. Procyanidins (PC) are beneficial compounds but have poor stability. Researchers used wheat starch aerogel (WSA) to encapsulate PC due to its higher loading capability and compatibility. The release rate reached 10.92%, 28.93%, and 55.69% with the modifications of starch. Most likely due to the addition of phosphate groups, which increased PC-WSA’s stability in the stomach fluid phase, PC-WSA released more PC in the SIF and comparatively less PC in the SGF. PC-WSA had a network structure with encapsulated PC and showed improved stability against high temperatures and light exposure. The study suggests starch-based aerogels have the potential to enhance the stability and application of PC, including drug delivery [80]. Through the use of emulsion–gelation processes and supercritical fluid extraction, García-González et al. have successfully produced microspherical aerogel particles for drug delivery systems [110]. The researchers created highly porous aerogels by optimizing the starch concentration and source. These starch gels were then templated into microspheres (400–800 um), forming highly porous aerogel microspheres with a substantial loading capacity for ketoprofen. The researchers found that the aerogel microspheres had a significant loading capacity for ketoprofen, reaching approximately 11 wt.% by supercritical impregnation. Following a two-step profile in aqueous solutions, it was freed from the starch aerogel matrix and mimicked physiological circumstances. The drug released up to 56% at the first 2 h and then showed sustained release up to 24 h. Another study added three non-steroidal anti-inflammatory drugs (NSAIDs) (nimesulide, ketoprofen, and diclofenac sodium) into maize starch aerogel (MSA) and calcium alginate aerogel (CAA) using SCCO_2_ adsorption. CAA had more drug loadings than MSA because of its higher surface area. NSAIDs adsorbed into MSA were released faster in comparison to the crystalline drugs, while CAA aided in a more controlled release of the drug. Nimesulide, ketoprofen, and diclofenac sodium absorbed within the MSA were released within 13 h, 1.47 h, and 1.2 h, respectively, while in CAA, they were released within 69 h, 2.7 h, and 7 h, respectively. This makes MSAs useful in creating drugs that use orally-based tablets, while CAAs are better for DDS methods that require prolonged release. The compositions of the drug loadings met pharmaceutical dosage requirements, highlighting the potential of aerogels in pharmaceutical applications [81]. Aerogel in the form of microspheres has been shown to be an effective carrier for drug delivery. In a study by Sang et al., diatomite-incorporated hydroxypropyl cellulose/chitosan (DE/HPC/CS) composite aerogel microspheres were created to deliver 5-fluorouracil (5-Fu) [111]. The goal was to improve drug encapsulation efficiency, release control, sustainability, and pH responsiveness. The resulting aerogel microspheres, which were approximately 0.5 mm in diameter, achieved a maximum encapsulation efficiency of 82.6% with optimal DE content. The release profiles of both DE-incorporated and Eudragit-coated microspheres were evaluated under varying pH conditions and analyzed using kinetic models. The results indicated that increasing DE content slowed the release rate of 5-Fu while extending its release duration.

### 3.5. Biopolymer-Based Aerogel for Biosensors

Aerogels with their porous structure, ultrahigh specific area, low density, and readily accessible adsorption sites, create great potential among 3D structural materials for usage in thermal insulation, energy storage, and wearable sensor applications. Using biopolymer-based aerogels for pressure sensors is an intriguing idea. Biopolymers, derived from natural sources, such as cellulose or silk fibroin, offer several advantages over synthetic polymers, including biodegradability, biocompatibility, and sustainability.

**Figure 6:**
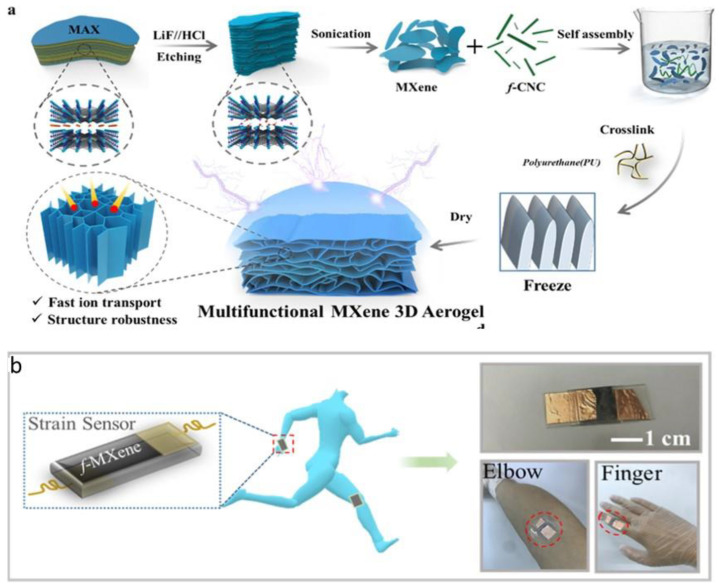
Examples of a Mxene/cellulose composite aerogel used for a biosensor. (**a**) Preparation process of MXene aerogels; (**b**) illustration and pictures of the prepared wearable MXene-based strain sensor [82].

A new method has been developed to create structured three-dimensional (3D) aerogels with controllable topography, using functionalized cellulose nanocrystals (f-CNC) as structure modifiers. By utilizing f-CNCs as a structural modifier and controlling the cryo-assembling process, structured MXene 3D aerogels with high MXene loading (85%) were achieved. Due to porous channels with multilevel nanostructured configurations, these aerogels exhibited high temperature-resistant mechanical superelasticity (70.4% stress retention under 100 cycles) and electrochemical properties (225 F/g at 2 mV/s). Symmetric MXene supercapacitors assembled from these aerogels demonstrated excellent energy density, cycle stability (86.7% after 4000 cycles), and superior photoresponse ability. The aerogel exhibited a highly sensitive response to bending, which may apply to wearable electronic devices monitoring human motions (Figure 6) [82]. The TEMPO–oxidate CNF (TOCNF)-rGO aerogels also showed great potential as pressure sensors. They are created using a combination of TEMPO cellulose nanofibers and reduced graphene oxide, which results in a lightweight, conductive, elastic, and sturdy aerogel. The aerogel’s tubular structure is reminiscent of natural wood, providing it with exceptional mechanical strength, compressibility, and rebound properties. It functions as a reliable pressure sensor, with high sensitivity to initial pressure (1.12 kP^−1^), quick response time (<100 ms), and excellent compression cycle stability (1500 cycles). The TOCNF-rGO was assembled successfully to capture human biological and motion signals. Elbow and knee joint joint motion was detected accurately, and the arterial pulse was recorded. Additionally, it showed excellent electrochemical properties in all-solid-state applications [112]. Another study involved the integration of cellulose-based aerogels with carbon nanotubes and nanofibers, resulting in hybrid aerogels that are ultralightweight, highly compressible, and fatigue-resistant. These innovative aerogels boast excellent electrical conductivity, making them ideal for use as pressure sensors and electrode materials in compressive solid-state supercapacitors. They are made using a bidirectional freezing strategy, which results in a tracheid-like structure with intertwined CNF and CNT “mortars” held together alongside MXene “bricks”. This biomimetic texture enables the aerogels to exhibit high compressibility, fatigue resistance, and low density, with a maximum of 80% compressibility and a high fatigue resistance of 1000 cycles at 50% strain. As pressure sensors, the aerogels demonstrated remarkable sensitivity, making them suitable for monitoring body surface information and detecting human motion [83].

Flexible and thin pressure sensors were created using cellulose aerogels of bacterial cellulose (BC) and silver nanowires (AgNW). These BC-AgNWs aerogels boasted impressive mechanical properties and high sensitivity (47.1 mA kPa^−1^ V^−1^). By sandwiching the aerogel films between PET films, the flexible pressure sensors offered a wide detection range, speedy response time (220 ms), a low detection limit of 400 pa, and exceptional stability over 5000 cycles. These sensors were ideal for detecting human movements and monitoring health conditions. In particular, the BC-AgNWs aerogel detected spinal muscle atrophy (SMA), which allowed patients to monitor their muscle atrophy effectively and receive prompt medical treatment. This study underscores the potential of BC-based aerogels in the realm of sensitive sensors [113]. Rod-shaped cellulose nanocrystalline (CNC) particles are a great alternative to micrometer-sized cellulose scaffolds and particles for the construction of functional materials in flexible electrodes and pressure sensors. Cellulose aerogels can be used to create graphene/CNC aerogel monoliths with a vein-like texture-mimicking natural structures. This material is ideal for wearable devices and electronic skins. The resulting materials exhibited high compressibility (up to 95% strain) and high sensitivity at a wide working pressure range of 0–57 kPa, thus showing good mechanical and electrical properties. Additionally, the sensor was also able to accurately detect human biosignals as well [114].

Silk-based aerogels are also used in pressure sensors. Abadi and colleagues successfully created lightweight and flexible wearable electronics using advanced composite foams. These foams were made using silk fibroin, graphene oxide (GO), and Ti3C2 MXene nanosheets by using a bidirectional freeze-casting technique, which resulted in nacre-like structures that are highly resilient, electrically conductive, and exceptionally lightweight. This breakthrough innovation has many potential applications, such as pressure sensing, oil/water separation, and mechanically switchable electronics. Furthermore, these composite foams have demonstrated remarkable sensitivity and performance as strain/pressure sensors with a detection range of up to 50% and pressure from ∼1 Pa to 2 kPa, making them ideal for flexible sensing arrays [84]. Another research team has produced an impressive range of biocompatible materials inspired by the catholic amino acid DOPA found in the mussel foot plaque. These materials, which include hydrogels, gums, and aerogels, are built on a foundation of silk fibroin, tannic acid (TA), and MXene nanosheets. They offer a variety of properties, such as elasticity, electrical conductivity, and mechanical strength. The materials possess remarkable stretchability, self-healing capabilities, adhesiveness, biocompatibility, and 6.5 × 10^−4^ S cm^−1^ electrical conductivity. The gum sensors were attached to a number of bodily joints, including the elbow, shoulder, knee, and finger. The joints were bent from their relaxed position to record variations in resistance. The potential of these materials for biomedical engineering, prosthetics, and other medical applications is promising. They can be used in various soft electronic devices, including electronic skins, piezoresistive wearable pressure sensors, health monitoring devices, and in soft robotics applications [115].

## 4. Conclusions and Perspectives

Biopolymer-based aerogels are gaining a lot of attention because of their special features, such as high porosity, a large surface area, ultra-low density, and biocompatibility. These aerogels’ high porosity and biocompatibility make them great scaffold materials for tissue engineering since they can promote cell adhesion, growth, and differentiation. Additionally, these aerogels can maintain a moist environment conducive to healing while absorbing excess exudates. The large surface area of biopolymer aerogels provides more active sites for the immobilization of sensing elements, increasing the sensitivity of biosensors. The porous structure also facilitates rapid diffusion of analytes to the sensing sites, improving response times. Furthermore, the mechanical properties of biopolymer aerogels can be adjusted to develop flexible and wearable biosensors that adhere to the skin or other tissues, providing continuous monitoring of health parameters. Despite all of this, there are several difficulties with aerogels. For instance, (i) biopolymer-based aerogels are naturally brittle and can fracture under stress, which hinders the promotion of tissue regeneration. Improving their potency without sacrificing any of their other advantageous qualities is still a major task. (ii) It is crucial that scaffolds break down at a pace corresponding to tissue regeneration; unfortunately, it can be difficult to create aerogels with predictable breakdown rates that do not result in hazardous byproducts.

To generate optimal aerogels for tissue regeneration, interdisciplinary research combining breakthroughs in biomedical engineering, materials science, and tissue biology is needed. Surface modifications can improve targeted delivery to specific tissues or cells, reducing side effects and improving treatment outcomes. Combining advances in flexible electronics with biopolymer aerogels can create integrated wearable systems for real-time health monitoring. Developing scalable and cost-effective manufacturing processes for biopolymer aerogels is crucial for their widespread adoption. Additionally, enhancing biopolymer aerogels’ mechanical and chemical stability under physiological conditions is necessary for long-term applications. It is believed that the field of biopolymer-based aerogels is still in its infancy and that not much research has been carried out in biomedical applications thus far, with the majority of investigations and tests conducted in labs rather than being used in clinical trials or achieving commercialization.

## Figures and Tables

**Table 2:** Advantages and disadvantages of different biopolymers as aerogel.

Biopolymer	Advantages	Disadvantages	Refs
Cellulose	-Biocompatibility and high porosity	-Moisture sensitivity and mechanically fragile -Highly hydrophilic, leading to swelling and loss of mechanical properties in wet environments	[62,63]
Chitosan	-Biocompatibility; antimicrobial and and hemostatic properties with high porosity -Generally nonbrittle	-Solubility and stability can be affected by pH changes, limiting their use in certain biological environments.	[64,65,66]
Starch	-Biocompatibility, biodegradability, high porosity, and cost-effectiveness	-Starch is highly hydrophilic, which can lead to moisture absorption, swelling, and structural instability in moist environments	[67,68]

**Table 3:** Overview of different biopolymer-based aerogels and their applications.

Aerogel Type	Fabrication Method	Properties	Applications	Refs
Silk fibroin/cellulose composite aerogel	Alkali/urea system and freeze-drying	Porosity: 99.2% Compressive strength: 7–10 MPa	Bone tissue regeneration	[69]
Methylcellulose-in-bacterial nanocellulose aerogel	3D printing and SC-CO_2_	Porosity: 84–87% Pore size: 19–25 nm Surface area: 268–283 m^2^/g Pore volume: 1.71–2.37 cm^3^/g	Bone tissue engineering	[70]
Silk fibroin aerogel	Self-assembly with photo-cross-linking-assisted 3D printing	Pore size: 7–23 μm	Treatment of bone cancer	[71]
Silk fibroin aerogel	Photo-crosslinking and 3D printing	Honeycomb-like pores of ≈100–120 µm with interconnected macropores (1000 μm) Surface area: 308.8 m^2^/g	Promoted osteoblastic differentiation	[72]
Starch aerogel microsphere	Emulsion mixture with thermal gelation process along with solvent exchange	Porosity: 87.7 ± 0.2% Surface area: 188 m^2^/g Pore volume: 1.25 cm^3^/g	Scaffolds for bone regeneration	[73]
Alginate–starch based aerogels	CO_2_-induced gelation followed by expansion of the dissolved CO_2_	Porosity: 25 ± 2%, Pore size of 431 ± 158 μm Surface area: 183–544 m^2^/g Pore volume: 2.0–6.8 cm^3^/g	Bone regeneration	[74]
Poly(ethylene glycol)diacrylate/cellulose nanofibril aerogel	Stereolithography and freeze drying	Young’s Modulus: 0.58 ± 0.02 or 0.51 ± 0.03 depending on wet or dry states and Poisson’s ratio of 0.08 ± 0.02 or 0.04 ± 0.002	Chondrogenic differentiation	[75]
Nanocellulose/PEGDA aerogels	Stereolithography and freeze-drying	Macropore sizes: 400 and 800 μm Micropore sizes: 20 to 100 μm	Cartilage repair in tissue engineering	[76]
Bacterial cellulose aerogels	Multiple freeze/thaw cycles and tert-butanol dehydration	Porosity: 97.35 ± 0.36% Pore sizes: 28.16 ± 9.58 to 2.56 ± 0.42 µm Surface area: 42.22 ± 8.21 m^2^/g	Ultra-fast hemostasis and wound healing	[77]
Cellulose aerogel loaded with turmeric-derived nanoparticles	Ice-templating, solvent exchange, and air-drying procedure	Water vapor transmission rate: 1967 ± 193 g (m^2^ per day)^−1^ Swelling ratio: ≈6000%	Diabetic wound healing	[78]
Starch–konjac glucomannan aerogel	Dispersion of microporous starch followed by freeze-drying methods	Porosity of 59.91 ± 5.4%, Pore size: size of 41 ± 7.3 μm	Hemostatic dressing that facilitates tissue repair of bleeding wounds	[79]
Wheat starch-based aerogels	Mixture of starch pasting with cooling alongside freeze drying	Pore size: 23.108 nm Surface area: 8.108 m^2^/g Pore volume: 4.684 × 10^−2^ cc/g	Drug delivery	[80]
Starch–alginate aerogel	Gelatinization and retrogradation followed by drying in SC-CO_2_	Pore size: 0.26 ± 0.27 to 0.13 ± 0.10μm Surface area: 58.3 ± 0.5 to 44.4 ± 0.7 m^2^/g	Anti-inflammatory drugs delivery	[81]
MXene nanosheets, waterborne polyurethane, and functionalized cellulose nanocrystal aerogels	Bioinspired freezing method	Thermal conductivity: 0.045 W m^−1^ K^−1^ Electrochemical property of 225 F/g and cycle ability of 86.7% after 4000 cycles	Integrated photodetector or biosensor for wearable electronics	[82]
MXene/nanocellulose composite aerogels	Stirring and ultrasonication of CNF/CNT/MXene with freeze drying	Electrochemical performance: (849.2 mF cm^−2^ at 0.8 mA cm^−2^ Ultralow density: 7.48 mg cm^−3^ Electrical conductivity: ~2400 S m^−1^	Pressure sensors and compressible electrodes	[83]
Silk fibroin–MXene composite aerogels	GO nanosheets and MXene in a cross-linked SF fibrous solution via bidirectional freeze-casting, followed by freeze-drying and gas-phase hydrophobization	Density: 6–60 mg cm^−3^ Electrical conductivity: up to 20,000 S cm^−1^)	Mechanically switchable electronics for flexible pressure sensors/electronic skins and pollutant separation	[84]
3D-printed alginate-hydroxyapatite aerogel	3D-printing of alginate-hydroxyapatite hydrogels combined with supercritical CO_2_ drying methodology	Porosity: 88.56 to 80.33% Surface area: 183 ± 9 m^2^/g Pore volume: 3.14 ± 0.16 cm^3^/g	Bone regeneration	[85]
PLLA/Gel/HA/CS gradient fibrous aerogel	PLLA/Gel/HA/CS fibers homogenized in butanol with NaCl particles followed by freezing in liquid nitrogen and freeze-dried for 24 h	Fiber diameter: approx. 2.6 μm Compressive strength: 1.4 MPa for the strongest variant.	Osteochondral tissue engineering in the way of no growth factor addition	[86]
Chitosan/gelatin/dopamine aerogel	Mixed the DA-grafted gelatin and chitosan solution followed by immersing in a EDC/NHS/ethanol solution overnight and freeze-dried	Pore size: 48.972 ± 18.172 μm Density: 25 mg/cm^3^ Water absorption rate: >2000%	Multifunctional graft for the treatment of chronic wounds	[87]
PGLA/gelatin and PDO/gelatin nanofiber aerogels	Freeze-casting technique	Diameters of macrochannels ≈ 300–500 µm	Treatment and fast healing of diabetic wounds	[88]

## Data Availability

Not applicable.
